# Development of a Web-Based Multimedia Patient Decision Aid for Rheumatoid Arthritis: A User-Centered Design

**DOI:** 10.3390/healthcare14080983

**Published:** 2026-04-09

**Authors:** Effie Simou, Dimitrios Tseronis, Konstantina Zoupidou, Dimitrios Boumpas

**Affiliations:** 1 Department of Public Health Policy, School of Public Health, University of West Attica, 11521 Athens, Greece; 2 Rheumatology and Clinical Immunology Unit, “Attikon” University Hospital, National and Kapodistrian University of Athens, 11527 Athens, Greece

**Keywords:** shared decision making, patient decision aid, digital tools, rheumatoid arthritis, web-based decision aids, health communication, digital health

## Abstract

**Background:** Shared decision-making (SDM) is particularly relevant in rheumatoid arthritis (RA), where multiple treatment options with distinct benefit–risk profiles require alignment with patient values and preferences. This study describes the development of a web-based PtDA to support treatment decision-making in RA and represents the first structured, standards-aligned PtDA in the Greek healthcare context. **Methods:** Guided by the Ottawa Decision Support Framework and the International Patient Decision Aid Standards, a multistage, user-centered methodology was applied, including evidence synthesis, iterative prototyping, and alpha and beta testing. Qualitative methods, including focus group discussions, semi-structured interviews, and think-aloud protocols, were used, while usability was assessed with the System Usability Scale (SUS). Methodological quality was evaluated using IPDASi v3 and UCD-11 criteria. **Results:** The final PtDA provides a three-step pathway supporting values clarification, comparison of medication options, and reflection on decisional confidence. It was developed as a publicly accessible, web-based tool compatible with multiple devices, with core elements also available in printable format. The tool showed good usability (mean SUS: 75.93) and strong alignment with IPDASi (83.3/100), and user-centered design criteria (11/11). **Conclusions:** Developing digital PtDAs is inherently complex, underscoring the importance of established methodological frameworks. The findings demonstrate acceptable usability and alignment with established standards within this early-stage development study. Further research is required to examine the tool’s impact on decision-making processes, value–choice concordance, and longer-term clinical outcomes.

## 1. Introduction

Person-centered care is a fundamental principle in contemporary healthcare, grounded in ethical responsibility and commitment to engaging patients as active partners in decisions about their care. Shared decision-making (SDM) provides a well-established conceptual framework for this practice by integrating clinical evidence, expertise, and patient values in the co-creation of treatment plans [1,2,3,4]. Although SDM is associated with increased patient knowledge and improved health outcomes [5,6], it remains inconsistently embedded in routine practice, particularly in hospital settings, where time pressure and organizational constraints limit its use [7,8,9,10]. The process is further influenced by clinicians’ communication skills, patients’ ability to comprehend information, and the absence of guidelines that formally integrate SDM into routine care [10].

Patient decision aids (PtDAs) are evidence-based tools designed to support SDM by providing balanced evidence on options and facilitating patient reflection on personal values [11,12]. PtDAs are consistently associated with enhanced patient knowledge of the risks and benefits of treatment options, reduced decisional conflict, and more effective patient–doctor communication, with no significant impact on anxiety levels or consultation duration [11,12,13,14]. Their development is typically guided by quality criteria, such as the Ottawa Decision Support Framework and the International Patient Decision Aid Standards (IPDAS), ensuring consistency and credibility [15,16,17,18,19]. Contemporary scholarship further emphasizes user-centered co-design approaches, positioning patients and health professionals as active contributors throughout iterative development processes [20,21,22]. Digital technologies have further expanded the potential of decision support beyond time-limited consultations. Digital PtDAs delivered through web platforms or mobile applications can incorporate interactive, personalized, and multimedia features that facilitate the understanding of complex medical information. They can also be integrated into electronic health record (EHR) systems, supporting their use in routine clinical workflows. However, barriers remain, including limited trust in digital tools, unequal access to technology or Internet connectivity, and variations in patients’ digital literacy [23,24].

Rheumatoid arthritis (RA) represents a clinical context in which SDM and PtDAs are particularly relevant, as clinicians and patients must navigate multiple therapeutic options, including conventional synthetic (csDMARDs), biologic (bDMARDs), and targeted synthetic DMARDs (tsDMARDs), such as Janus kinase (JAK) inhibitors, each characterized by distinct benefit–risk profiles and monitoring requirements [25]. Consequently, SDM is recognized as a core principle within contemporary RA treatment guidelines and “treat-to-target” recommendations [26,27]. Despite strong evidence supporting SDM and PtDAs, their routine integration into rheumatology practices remains limited. This is partly due to structural and organizational challenges, including limited time, complex treatment decisions, and difficulties in aligning care with patient-centered priorities, as well as variability in health literacy, system-level constraints, and limited clinician familiarity with these tools [28,29]. Recent reviews have highlighted additional methodological challenges [30,31]. Although SDM interventions are associated with improved patient knowledge, satisfaction, and decisional confidence, substantial heterogeneity in outcome measures and contextual factors limits comparability across studies [30]. Similarly, existing PtDAs vary considerably in terms of development approaches, reporting transparency, and evaluation methods, reflecting a fragmented and evolving field [31].

Significant gaps persist at the national level, as no PtDAs specifically developed for the Greek healthcare context have been identified to date. A targeted search of PubMed, Scopus, and Google Scholar did not identify any Greek-language tools designed to support medication decision-making for RA. To address this gap, we developed a web-based PtDA designed to provide a structured comparison of all available treatment options for RA, while supporting patients in aligning these options with their individual preferences and values. The objectives of this study were to: (i) design the PtDA in accordance with established frameworks, (ii) iteratively refine the tool based on feedback from patients and clinicians, and (iii) conduct an initial usability evaluation with end-users.

## 2. Materials and Methods

### 2.1. Study Design and Conceptual Framework

**Figure 1:**
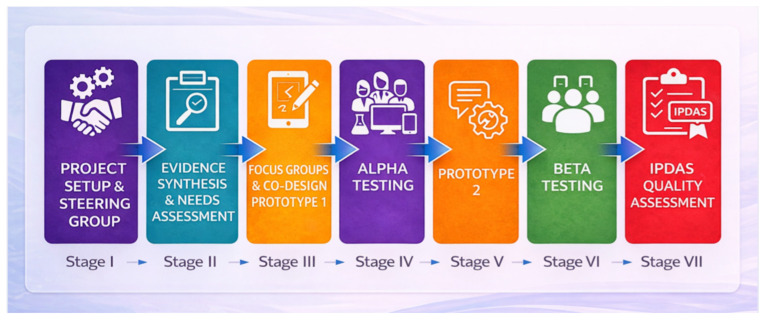
Seven-stage development and initial evaluation of the web-based PtDA.

This project was guided by the Ottawa Decision Support Framework [32] and the International Patient Decision Aid Standards (IPDAS) [33], and was conducted between September 2021 and May 2022. User involvement was a central methodological commitment, with all stages documented in accordance with the DEVELOPTOOLS checklist and UCD-11 scale [22]. As illustrated in Figure 1, the process followed a structured, user-centered pathway comprising seven stages: (I) project setup and establishment of a multidisciplinary steering group to define scope and purpose, (II) evidence synthesis and needs assessment to identify key content and layout requirements, (III) co-design through focus groups with patients and clinicians, leading to the development of Prototype 1, (IV) alpha testing to evaluate content, comprehensibility, usability, and acceptability of the tool’s format (including text, audio, images, and animated video), (V) refinement and development of Prototype 2, (VI) beta testing to assess usability in a clinical context, and (VII) final quality assessment of the completed PtDA using IPDAS criteria to ensure alignment with established standards. Data were generated through focus group discussions [34] and individual interviews [35], incorporating the think-aloud technique [36], allowing participants to articulate their thoughts while interacting with the PtDA. The data were analyzed using reflexive thematic analysis [37,38], with data collection in each phase continuing until thematic saturation was reached [39]. Written informed consent was obtained from all participants.

### 2.2. Project Setup, Scope and Steering Group

A multidisciplinary steering group was established at the beginning of the development process, comprising a professor of rheumatology, a patient with RA, a rheumatology nurse, and an associate professor in health communication and public health. The project was conducted under the auspices of the Greek Rheumatology Society and the Greek Association of Professional Rheumatologists, and the Panhellenic Federation of Associations of Patients, Parents, Guardians, and Friends of Children with Rheumatic Diseases (REUMAZIN). The steering group was actively involved throughout the development process, providing ongoing guidance and reviewing drafts and prototype mock-ups before final implementation.

The PtDA was developed to support patients with moderate-to-severe RA in better understanding and comparing the available pharmacological treatment options. Designed as a web-based tool accessible across devices, it can be used before, during, and after clinical consultations. By offering evidence-based information alongside opportunities for reflection, the tool aims to help patients make informed decisions that align with their values, while also supporting more meaningful discussions with clinicians, without replacing the clinical encounter.

### 2.3. Literature Review

The literature review informed both the content and functional design of the PtDA integrating evidence across three domains: (i) the availability of existing PtDAs for RA, (ii) patient and clinician preferences, including barriers and facilitators shaping decision-making in clinical practice, and (iii) the efficacy and safety of RA treatment options based on the best available evidence. Searches were conducted between September and October 2021 in PubMed, Embase, and the Cochrane Library, complemented by grey literature from professional organizations. Inclusion criteria covered existing PtDAs and evidence from randomized controlled trials, systematic reviews, meta-analyses, and clinical guidelines. Only English-language publications were included, while studies focusing on specific subpopulations were excluded to enhance generalizability. Existing decision aids, including the A-to-Z Inventory of Decision Aids, were also reviewed to inform the PtDA’s structure, layout, and content [40]. Findings were synthesized analytically, with attention to strengths, limitations, and implications for design. Based on methodological quality, relevance to RA treatment decisions, and alignment with IPDAS criteria, five tools were identified as key sources of inspiration [41,42,43,44,45]. This review was not intended as a formal systematic review, but as a focused, design-oriented synthesis to support content development and alignment with established decision aid standards.

### 2.4. Focus Groups

Following a literature review, a qualitative needs assessment was conducted via focus groups [34] at Attikon University General Hospital to explore how treatment decisions are experienced in routine RA care. Four focus groups were conducted: two groups of patients (n = 6 per group; four women and two men; mean age 52.0 years) and two groups of clinicians (n = 6 per group; four women and two men; mean age 47.0 years). Patients and clinicians were recruited through convenience sampling from the hospital’s outpatient rheumatology clinics. Each group consisted of the same participants across two iterative sessions, allowing for continuity and deeper exploration of emerging themes. This approach enabled the refinement of insights through a structured, iterative process. The choice of six participants per group was purposeful, balancing the need for deep, focused discussion with the benefits of participant homogeneity [46]. Qualitative research suggests that groups of 4–6 are ideal for exploring complex topics, facilitating balanced interaction and the detailed refinement of tools like a PtDA [46,47]. The use of the same participants across two rounds also supported continuity, enabled more in-depth exploration of emerging issues, and informed the iterative refinement of the prototype. A complete set of focus group questions and prompts is provided in Section S1.

The qualitative analysis of the focus group data followed a reflexive thematic approach, with careful attention to preserving the distinct perspectives of patients and clinicians [37,38]. Audio-recorded discussions and field notes were reviewed in depth and coded at the semantic level to capture recurring patterns in decision-making experiences and informational needs. To retain the specificity of each perspective, patient and clinician datasets were initially analyzed separately and subsequently compared to identify areas of convergence and divergence. Theme and coding development were conducted collaboratively, with differences resolved through discussion. The analysis was interpretive, with researchers actively engaged in the interpretation of the data. Rather than treating data saturation as a fixed or purely objective endpoint, data analysis proceeded iteratively until sufficient depth and conceptual clarity were achieved, and no new content -relevant insights or meaningful variation in participants’ perspectives were identified [38,39].

The analysis highlighted the complexity of decision-making in RA care. Patients emphasized the role of personal values, daily routines, and future expectations, while also drawing attention to less visible factors such as fatigue, emotional burden, and concerns about side effects, which shape adherence and trade-offs between short- and long-term outcomes. Clinicians, in turn, highlighted everyday constraints in routine care, such as limited consultation time, challenges in monitoring treatment, and gaps in available information that can make it harder for patients to engage in shared decision-making. These insights helped shape the content, structure, and intended use of the PtDA, ensuring that it reflects not only patient priorities but also the practical realities of clinical care.

### 2.5. Prototype Design, Content Development, and Web Design

The prototype was developed through an iterative process consisting of three stages: (i) the application of IPDAS quality criteria, (ii) the integration of evidence from a literature review and focus group discussions, and (iii) collaboration between graphic designers and a health communication expert to ensure a clear and user-friendly presentation of scientific evidence. The PtDA is structured as a three-step interactive pathway, which includes clarifying values and preferences, comparing treatment options, and assessing decisional confidence. The prototype incorporates seven pharmacological treatment options: methotrexate, leflunomide, hydroxychloroquine, non-steroidal anti-inflammatory drugs, glucocorticoids, biologic agents, and Janus kinase (JAK) inhibitors. These options were selected based on their inclusion in current RA treatment guidelines [25] and their availability within the Greek healthcare system, ensuring comprehensive coverage of all relevant and accessible treatment options.

Patient narratives [48] were incorporated to support reflection on personal concerns and uncertainties by drawing on the experiences of individuals with similar symptoms. Narratives were derived from qualitative data collected during focus group discussions. All participants provided written informed consent for the use of anonymized excerpts for research and educational purposes, in accordance with the ethical approval obtained for the study. Two audio-visual modules were created to improve knowledge translation, especially for individuals with lower health literacy [49]. The scripts were developed iteratively based on literature and qualitative findings, narrated in Greek by a professional voice actor, and reviewed by the steering group to ensure accuracy and consistency with evidence-based information. The PtDA also includes a communication support section that provides practical guidance to help patients prepare for consultations and effectively express their concerns and preferences.

A layered information architecture was employed to support a clearer understanding of complex health information. By presenting core messages first and allowing users to explore deeper details through expandable sections and guided pathways, we aimed to reduce cognitive load and facilitate clearer processing [50]. We also combined text with visual graphics to help users better interpret risks and probabilistic data, as visual aids often influence decision-making more effectively than numbers alone [51,52].

### 2.6. Alpha Testing

The aim of the alpha testing phase was to explore the comprehensibility, acceptability, and usability of the prototype from both patient and clinician perspectives. A total of 12 participants took part, including six patients (four women and two men, mean age: 57.7 years) and six clinicians (three women and three men, mean age: 49.0 years). Participants attended evaluation sessions in which they reviewed both paper-based and web-based mock-up versions of the PtDA. Recruitment was based on convenience sampling from outpatient rheumatology clinics at Attikon University General Hospital, ensuring that participants closely matched the target end-users in routine clinical practice. This approach is appropriate for early-stage usability testing, where the aim is to identify key usability issues and decision-support needs rather than achieve statistical representativeness. The sample size was considered adequate, as qualitative research suggests that thematic saturation is often reached with 6–12 participants in relatively homogeneous groups [53]. A descriptive qualitative design was employed, combining semi-structured interviews [35], think-aloud protocols [36], and the teach-back method [54] to support in-depth exploration and iterative refinement of the prototype.

During the sessions, participants were encouraged to verbalize their thoughts while interacting with the prototype. To support the conceptual focus of the alpha testing phase, established decision-support instruments (e.g., DCS, SURE, PrepDM) were used solely to inform the thematic structure of the qualitative interview guides. These tools were not administered as standardized quantitative scales, instead, their underlying items guided the semi-structured interviews and think-aloud protocols, supporting a richer understanding of participants’ experiences.

The evaluation of information balance, adequacy, usability, and overall acceptability was guided by elements of the Ottawa Acceptability Tool (OAT) [55], including clarity, ease of navigation, and perceived usefulness. Participants were asked whether the information was balanced, the content length appropriate, the tool easy to navigate, and the visual presentation clear, as well as whether they found it useful and would recommend it to others. To explore decisional uncertainty, informed choice, and value clarity, we drew on constructs from the Decisional Conflict Scale (DCS) [56], focusing on participants’ understanding of available options and their associated benefits and risks. Participants also reflected on whether uncertainty decreased, options became clearer, decisions aligned with their values, and confidence in decision-making improved. The Preparation for Decision Making (PrepDM) scale [57] informed questions exploring how participants perceived the role of the PtDA in supporting the decision-making process, including clarifying values, weighing benefits and risks, and preparing for clinical consultations. Participants further considered whether the tool helped them identify priorities, compare options, formulate questions, and feel ready to engage in discussions. Finally, elements from the SURE scale [58] were incorporated to assess decisional certainty and perceived support. The complete semi-structured interview guide is provided in Section S2.

**Figure 2:**
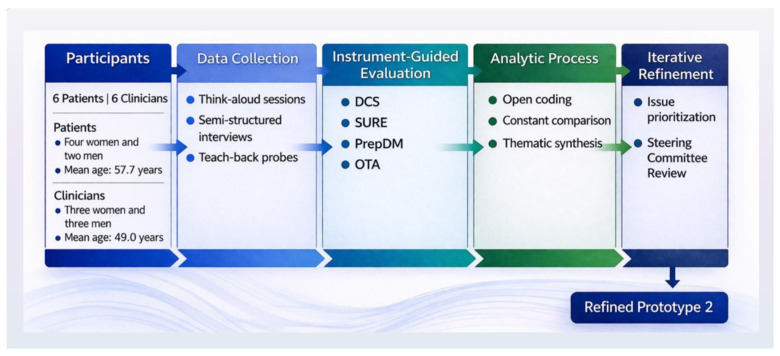
Structured Alpha Testing and Iterative Refinement Framework.

Participant feedback was documented directly on the prototype by the health communicator. The qualitative analysis of the alpha testing interviews was conducted using a reflexive thematic approach [37,38], with the aim of generating practical insights into content and usability. Data were reviewed iteratively and coded based on what participants directly expressed, capturing recurring issues related to comprehension, navigation, information balance, and decision support. Coding was conducted inductively, allowing patterns to be grounded in participants’ experiences, followed by the systematic grouping of codes into broader categories reflecting key usability and design dimensions. These categories were subsequently refined into themes through an iterative process of comparison, discussion, and interpretation within the research team. In contrast to the focus group phase, the analysis at this stage was more applied and design-focused, with an emphasis on identifying specific issues that could directly inform improvements to the prototype. Coding was primarily undertaken by one researcher and subsequently reviewed by a second, with regular discussions used to refine interpretations and enhance consensus. Saturation continued until no new usability issues, content-related concerns, or design implications were identified, indicating that the data were sufficiently rich to support meaningful refinement of the tool [39,59]. These findings informed iterative refinements, coordinated between the health communicator and the software developer, resulting in Prototype 2. Figure 2 illustrates the structured alpha testing and iterative refinement framework, combining qualitative data collection, instrument-guided evaluation, and thematic analysis to inform the development of the refined prototype. 

### 2.7. Beta Testing

A beta testing evaluation was conducted to assess the usability of the PtDA under routine clinical conditions. Participants were recruited through convenience sampling from Attikon University General Hospital and included 10 patients with RA attending outpatient rheumatology clinics and 7 clinicians practicing within the same setting. Among patients, 70% were female (n = 7) and 30% male (n = 3), with a mean age of 52.4 years. Among clinicians, 57.1% were female (n = 4) and 42.9% male (n = 3), with a mean age of 44.6 years. Participants were invited to interact with the PtDA in a manner reflecting routine clinical use. Patients accessed the tool either immediately before or after their consultation, depending on clinic flow, whereas clinicians interacted with the tool independently within the context of their routine clinical practice. Brief, standardized instructions were provided to ensure basic orientation, while avoiding extensive training that could artificially influence usability perceptions.

Usability was assessed using the SUS, a validated 10-item instrument employing a 5-point Likert scale (1 = strongly disagree to 5 = strongly agree) [60,61,62]. Item responses were scored according to the standard SUS scoring procedure, including reverse scoring of negatively worded items, and transformed to a 0–100 scale with a higher score indicating more user-friendly. The sample size for usability testing was informed by findings from a usability study, which suggests that most usability issues are identified early in the testing process, while the addition of further users leads to a progressively smaller number of new findings. Specifically, analyses demonstrate substantial variability with small samples, as groups of five participants may identify between 55% and 99% of usability problems, whereas increasing the sample to ten and twenty users raises minimum detection rates to approximately 80% and 95%, respectively [63].

### 2.8. Quality Assessment of the PtDA

The quality of the PtDA was assessed using IPDASi v3 [64], which comprises 47 items across 10 domains. Items related to DST—evaluation dimension—were not included in the scoring, as these require empirical evidence of effectiveness (e.g., improvements in patient knowledge and values–choice concordance), which falls beyond the scope of this early-stage, user-centered development study. In addition, items specific to test-based decision support were excluded, as they apply to diagnostic or screening contexts, whereas the present PtDA focuses on treatment decision-making in RA. Consequently, the assessment was conducted on 36 applicable items. Each item was rated on a 4-point Likert scale (1 = strongly disagree to 4 = strongly agree). Two independent reviewers evaluated all items separately, and any discrepancies were resolved through discussion and consensus, with no remaining disagreements. Prior to scoring, calibration discussions were conducted to ensure a shared understanding of scoring criteria. In line with this consensus-based approach, inter-coder reliability statistics were not calculated [65]. Total scores were obtained by summing item ratings and subsequently transformed to a standardized 0–100 scale using the formula:

$$\frac{\mathrm{observed}\;\mathrm{score}-\mathrm{minimum}\;\mathrm{possible}\;\mathrm{score}}{\mathrm{maximum}\;\mathrm{possible}\;\mathrm{score}-\mathrm{minimum}\;\mathrm{possible}\;\mathrm{score}}\times 100$$

User involvement in the development process was assessed using the UCD-11 scale [22]. Each criterion was rated as fulfilled (1) or not fulfilled (0), based on documented evidence from the development process.

## 3. Results

### 3.1. Alpha Testing and Prototype 2 Refinement with User Feedback

The detailed findings from alpha testing are presented in Table 1.

#### 3.1.1. Patients Assessment

Patients generally perceived the PtDA as clear, informative, and easy to navigate, describing the content as “clear,” “well written,” and “informative”. Several (n = 5/6) reported that presenting treatment options side by side improved their understanding and highlighted the importance of making timely decisions. As one participant noted, “Seeing everything together made me realize that doing nothing is also a decision.” Participants valued the integration of clinical information with practical considerations relevant to their everyday lives. Most (n = 5/6) emphasized the importance of factors such as route of administration, frequency, time to effect, and contraindications when comparing treatment options. However, a subset of participants (n = 3/6) found the detailed presentation of side effects overwhelming and noted that the decision aid contained an excessive amount of text. The values clarification component supported reflection, with several participants (n = 4/6) reporting that it helped them identify previously unarticulated priorities, such as maintaining independence and quality of life. However, a small number (n = 2/6) initially experienced difficulties in translating these reflections into concrete treatment decisions. Visual elements and videos were well received (n = 5/6), contributing to improved understanding and engagement of the participants.

#### 3.1.2. Clinician’s Assessment

Clinicians evaluated the PtDA positively in terms of structure and clarity, with most (n = 5/6) considering it clinically appropriate and supportive of shared decision-making, describing it as “interactive and convenient.” Several (n = 4/6) noted that the tool could facilitate more focused consultations by helping patients to clarify their preferences in advance. Some concerns were raised regarding the potential impact on consultation time (n = 2/6 physicians). While usability was generally rated positively, a few clinicians (n = 2/6) identified minor navigation challenges, particularly in the treatment comparison section. Clinicians also emphasized the importance of integration into routine practice, highlighting features such as printable summaries (n = 4/6) as being particularly useful. Educational videos were consistently well received (n = 6/6), with clinicians noting their value in enhancing patient understanding, particularly among individuals with lower health literac.

#### 3.1.3. Prototype 2 Refinement with User Feedback

Following alpha testing, Prototype 1 was refined to improve its deliberative flow in response to end-users’ feedback. Participants emphasized the need for a clearer structure to support meaningful comparison and reduce cognitive burden. Accordingly, content was reorganized into coherent thematic sections, guiding users from understanding RA to identifying preferences and comparing alternatives. A redesigned decision pathway within the values clarification module, supported by a structured “Questions to Consider” section, strengthened the link between patients’ values and treatment trade-offs aimed to support patients in situating treatment decisions in the context of everyday experiences, moving beyond risk–benefit information toward more personal and context-sensitive decision-making. Key refinements focused on enhancing content clarity and risk communication. Terminology was simplified, and a layered information structure was introduced to enable progressive access to information. As extensive side-effect lists were perceived as overwhelming, risk presentation was standardized using natural frequency formats and focused on common side effects. An introductory statement clarified the general nature of risk information and the importance of clinician–patient discussion. To support values clarification and decision confidence, a reflection checkpoint—“How confident do I feel about this decision?”—was incorporated, maintaining a non-directive approach. Usability improvements included simplified navigation functionality and clearer section flow. Multimedia content was optimized through shorter videos with subtitles, enhancing accessibility. In addition, a full printable version was developed, alongside PDF export and email functionality, supporting a flexible dual digital–print format. Overall, Prototype 2 reflects the systematic integration of alpha testing findings into targeted design improvements.

### 3.2. Content of Final PtDA

**Figure 3:**
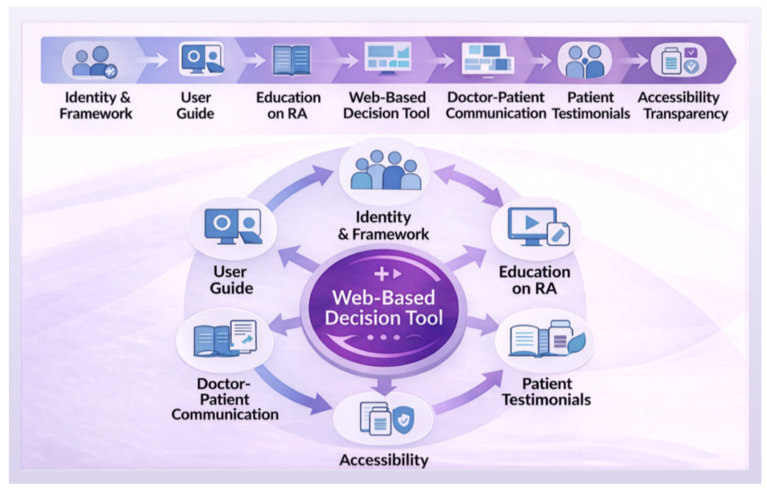
Sitemap of the Web-Based PtDA for RA.

The web-based PtDA was launched on 18 May 2022 during a press conference attended by representatives of the scientific community, clinicians, and patients, and is available at www.decisionaid.uniwa.gr. A print-optimized version of its core elements is also provided for users who prefer paper-based materials. The PtDA begins with a front page that introduces the program’s branding, “Together We Decide,” and situates the tool within an SMD framework for RA treatment. It clearly states its purpose as supporting, rather than replacing, medical consultation, while presenting the principles of SDM and offering educational content on RA, including two instructional videos. The decision tool is structured into three interactive modules: values clarification, treatment comparison, and decision confidence assessment. These modules are designed to support patient reflection on personal priorities, facilitate comparison of treatment options, and assess readiness to make a decision. The process concludes with a communication support component that provides structured guidance for patient engagement with clinicians before, during, and after consultation, thereby promoting informed and collaborative decision-making. The platform also includes a section with patient testimonials describing the lived experience of RA, addressing emotional, social, and practical aspects, and helping patients relate the content to their own circumstances. The platform is compatible with desktops, tablets, and mobile devices to ensure broad accessibility. It is compliant with the General Data Protection Regulation (GDPR) [66], does not require account creation, and stores session data only temporarily, which is automatically deleted after use, and does not connect to electronic health records or external databases. Figure 3 presents the structure and principal components of the PtDA.

More specifically, the core of the decision support tool begins with the section “Questions to consider when making a decision” and is organized into three subgroup components: (i) “Myself and rheumatoid arthritis” (assessment of symptoms and disease impact); (ii) “What is most important to me?” (clarification of values and preferences); and (iii) “Other factors influencing my decision” (contextual and clinical considerations). Together, these components support a comprehensive evaluation of decision-making needs, moving from patients’ personal experience of the disease (e.g., maintaining the ability to walk without pain, engaging with loved ones without discomfort, sustaining meaningful social relationships) to their values and preferences (e.g., preferences regarding mode of administration, such as oral medication versus injections, or receiving treatment in a hospital setting), and finally to relevant contextual and clinical factors, including comorbidities, history of serious side effects from previous treatments, pregnancy status, access to care, and lifestyle constraints (e.g., frequent travel), which may influence treatment decisions.

The second stage provides a side-by-side comparison of therapeutic options—methotrexate, leflunomide, hydroxychloroquine, non-steroidal anti-inflammatory drugs, glucocorticoids, biologic agents, and Janus kinase (JAK) inhibitors—using clear, accessible language to present key features, including route of administration, expected benefits, potential side effects, contraindications, practical implications for daily life, and combination options. Information is organized into structured comparison cards to support direct evaluation of treatments. Each card presents core dimensions, including mode and frequency of administration (e.g., oral, subcutaneous, intravenous; daily or weekly), expected benefits in both quantitative and plain-language formats (e.g., proportion of patients achieving improvement, time to onset), and common side effects. The side effects card summarizes commonly reported effects using plain-language descriptions, alongside a visual risk communication element (icon array), enhancing understanding of likelihood. Introductory guidance encourages patients to discuss the full range of side effects and their individual risk profiles with their physicians. Safety-related information is presented through clearly defined contraindications and conditions requiring caution, including (i) comorbid conditions (e.g., hepatic or renal impairment, severe infections, and visual or metabolic disorders) and (ii) lifestyle and contextual factors (e.g., pregnancy, breastfeeding, vaccination, and alcohol use). Finally, the module includes visual representations of combination therapy, illustrating how treatments can be used alone or in combination in routine clinical practice.

**Figure 4:**
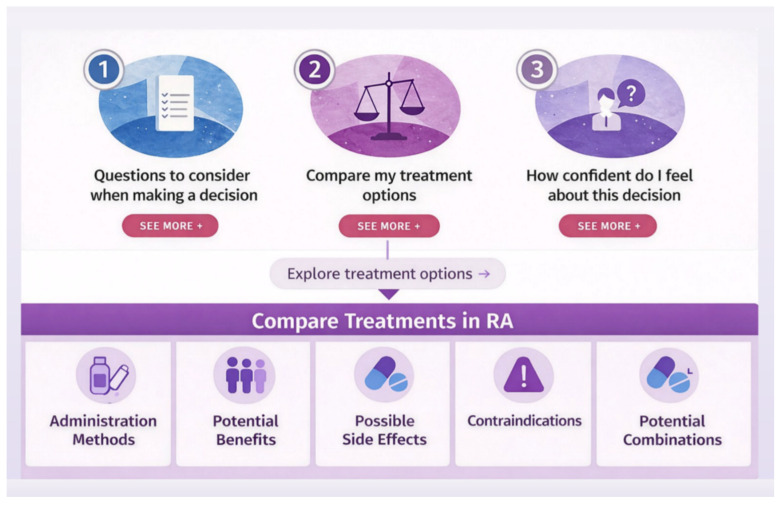
Functional core of the digital PtDA.

The final stage, “How confident do I feel about this decision?”, assesses decisional certainty through reflective questions regarding knowledge, confidence, and readiness. Patients evaluate their understanding of risks and treatment options, as well as their preparedness for clinical discussions and perceived support in decision-making. Users reflect on whether they feel ready to proceed or need further discussion with their physicians or others. Upon completion, users can generate a printable PDF summary of their responses for personal use or send their results via email to their physician or another recipient. Figure 4 illustrates the overall structure and decision-making flow of the web-based PtDA.

**Figure 5:**
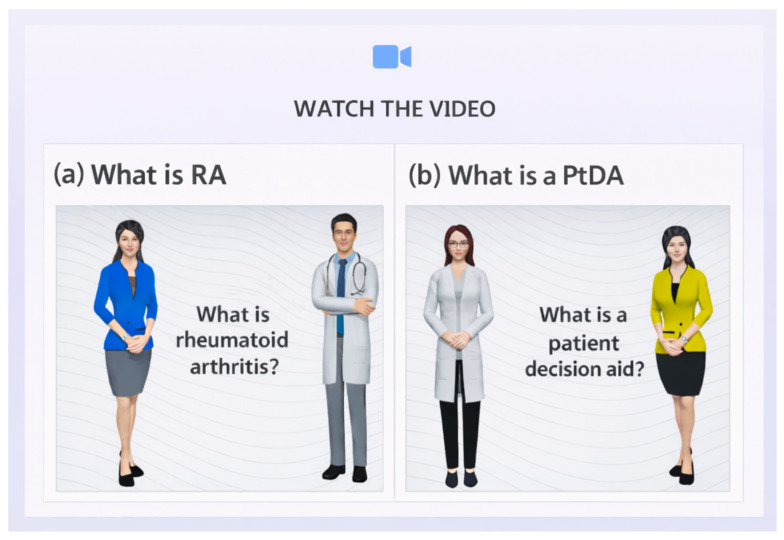
Representative screenshots of the educational videos incorporated into the web-based PtDA platform.

Figure 5 presents screenshots of two short educational videos developed for the web-based PtDA, which are also available on YouTube. The videos communicate key concepts in a clear and accessible format, particularly for patients with lower health literacy. The first video provides an overview of RA (duration: 2:48), while the second explains the purpose of the decision aid and offers step-by-step guidance on its use and preparation for clinical consultations (duration: 2:09).

### 3.3. Beta Testing

The prototype achieved a mean SUS score of 75.93, indicating good usability [61,62,63]. Patients reported slightly higher scores (77.25) than clinicians (74.00), however, these differences are descriptive and should be interpreted cautiously given the limited sample size. Overall, the findings suggest that the tool was well accepted by both groups. Detailed item-level results are presented in Table 2.

### 3.4. Quality Assessment and User-Centered Design Reporting

#### 3.4.1. IPDASi v3 Quality Assessment

The PtDA demonstrated high methodological quality, achieving a total score of 126 out of 144 (mean item score 3.50/4), corresponding to a standardized IPDASi score of 83.3/100. Of the 36 IPDASi items assessed, 28 received the maximum score of 4, indicating strong adherence to most quality criteria. Lower scores were primarily associated with formal reporting elements rather than core aspects of the tool’s conceptual design or functionality. For example, outcome probabilities were presented without explicit specification of the reference class, and no formal information on uncertainty (e.g., ranges or confidence estimates) was provided. Although the tool was informed by relevant literature, explicit study-level citations and a formal update policy were not systematically documented on the website, and readability was not assessed using standardized metrics. Overall, the pattern of scores indicates strong alignment with IPDASi quality standards, with lower ratings concentrated in reporting domains rather than in the core informational and decisional support components. A detailed item-by-item scoring justification is provided in Section S3.

#### 3.4.2. User-Centered Assessment

The development process followed a User-Centered Design (UCD) approach, fulfilling all 11 criteria for stakeholder involvement and iterative development. The implementation of the UCD-11 and DEVELOPTOOLS criteria is summarized in Table 3.

## 4. Discussion

This study reports the systematic development, iterative refinement, and preliminary evaluation of a web-based PtDA for RA, grounded in the Ottawa Decision Support Framework and aligned with IPDAS quality standards and UCD-11 criteria. The PtDA encompasses the full range of pharmacological treatment options currently available for RA within the Greek healthcare context. To our knowledge, it represents the first methodologically grounded and publicly available PtDA designed to support SDM in this setting. The development and implementation of web-based decision aids involve a range of technical challenges that require careful planning and implementation. The present contribution is both technical, by offering a user-friendly and accessible digital platform, and methodological, by demonstrating how SDM can be effectively implemented in a context where decision aids have been largely absent.

The study underscores that design extends beyond a purely technical task. In RA, treatment decisions are inherently complex, involving options that differ in risks, administration, monitoring, and impact on daily life. The challenge, therefore, is not only to present information, but to make it clear, comparable, and meaningful for patients. The PtDA addresses this through a three-part structure—values clarification, treatment comparison, and decisional confidence—supporting a more reflective and engaged decision-making process. This is particularly important in RA, where decisions rarely reduce to simple risk–benefit trade-offs and instead require balancing symptom control with side effects, treatment burden, and everyday priorities. At the same time, this approach invites critical reflection. The alpha testing findings provide valuable insights into how users engage with complex treatment information. The side-by-side comparison appeared to shape patients’ understanding and evaluation of their options. Several patients reported that seeing all treatments together, including the consequences of no treatment, influenced their decision to initiate, continue, or reconsider therapy. While this suggests a potential framing effect, the present data do not allow firm conclusions regarding the extent or nature of this influence. In decision theory, framing effects play a central role in how individuals interpret information and form choices. Prospect theory further shows that the way options are presented can influence risk appraisal and behavioral intentions, even when the underlying information remains unchanged [67,68]. Framing in decision support is therefore not only a matter of information processing, but also of how information is deliberately structured and prioritized, and must be carefully considered in both the design and presentation of scientific information.

The value clarification module was conceptually significant. Structured prompts helped participants articulate priorities, such as preserving independence, maintaining social roles, or addressing fears of disease progression, which were previously implicit. However, several participants struggled to translate their priorities into concrete trade-offs. This challenges the assumption that preferences are stable and readily articulated, suggesting they may evolve through reflection, while decisional confidence does not necessarily indicate value concordance. A patient may feel certain about a choice that does not fully align with their previously expressed priorities. Given the limited qualitative sample, this finding should be interpreted with caution. Therefore, further empirical assessment of the concordance between the stated values and selected treatments is required.

Additionally, although the PtDA was described as informative and well-structured, information-heavy sections, particularly extensive lists of side effects, reduced clarity and increased anxiety about long-term implications. This highlights that informational completeness does not guarantee cognitive accessibility. While the emphasis on common side effects was intended to enhance usability and reduce cognitive burden, it may reduce the visibility of rare but clinically significant adverse events. To mitigate this risk, the tool includes introductory guidance directing patients to discuss their individualized risk profile with their clinician, ensuring that rare yet important safety considerations remain part of the clinical encounter. The decision to focus on commonly reported side effects, presented using plain-language descriptions and visual formats such as icon arrays, is supported by evidence indicating that extensive lists—particularly when communicated through qualitative descriptors alone (e.g., “rare” or “very rare”)—can lead patients to overestimate risk [69]. Prioritizing common and clinically relevant side effects, and presenting them in visual formats, helps reduce cognitive load, improve risk interpretation, and mitigate anxiety related to low-probability but serious events. In this way, visual frequency formats provide essential context that verbal descriptors often lack, enabling patients to better understand risk without discouraging treatment engagement. These observations should also be considered in light of broader evidence on risk communication and decision support design [70]. A recent systematic review [71] showed that the effectiveness of PtDAS depends not only on the accuracy of information but also on how it is presented. Verbal descriptions may be misinterpreted, while numerical formats can be difficult to process, particularly for individuals with limited numeracy. Visual formats, especially when combined with numerical information, improve understanding and risk perception. Accordingly, the present PtDA integrates both formats. However, the findings highlight a tension between informational completeness and cognitive accessibility, particularly in relation to the extensive presentation of side-effect information. This reinforces the need for structured, risk communication approaches that enhance interpretability and ensure that patients are clearly and timely informed about potential risks, while avoiding unnecessary concern.

### 4.1. Usability Assessment

The PtDA achieved a mean SUS score of 75.93, indicating good usability. As the SUS provides a single composite measure of perceived usability rather than distinct subscales, the analysis focused on the aggregate score as a robust and widely accepted indicator [62]. Given the small sample size (n = 17), no inferential statistical comparisons were conducted, in accordance with the recommendations for early-stage evaluations. Item-level and subgroup results are presented descriptively to offer additional insight into user perceptions, but should be interpreted cautiously and not as independent dimensions of usability. While usability is a prerequisite for effective engagement with decision support tools, it does not ensure improved decision quality or value–choice concordance, which are more directly addressed by IPDAS criteria and longer-term assessments.

### 4.2. Quality Assessment

The tool demonstrated strong alignment with quality criteria and full compliance with user-centered design standards, while lower-scoring domains were primarily related to reporting and documentation rather than the intervention’s conceptual structure. This pattern is informative, suggesting that the core decisional architecture of the PtDA is methodologically sound, while highlighting areas for improvement in future iterations. Compared with previous studies, our findings show both convergence and important distinctions. For example, the patient-centered PtDA for type 2 diabetes [65] reported that 33 of 45 applicable IPDAS criteria were met (73%), with limitations primarily related to the absence of precise outcome probabilities, reflecting challenges in quantifying treatment effects in complex chronic conditions. Similarly, the decision aid developed for organized breast cancer screening [72] achieved very high construct quality (176/188; 43/47 items). Areas with relatively lower IPDASi scores—specifically, publicly documented evidence-based procedures and formal readability metrics—should be interpreted as opportunities for enhanced transparency rather than conceptual weaknesses. These limitations primarily reflect the practical challenges of applying multiple established analytical standards within an initial effort to translate and implement such tools in the Greek context. More explicit documentation of evidence sources, update procedures, readability assessment, and probability framing would improve transparency and reproducibility. As PtDAs increasingly move into digital and publicly accessible formats, transparency about evidence provenance and maintenance becomes part of implementation quality.

### 4.3. Methodological and Conceptual Contributions

The study also makes a useful contribution by documenting a full user-centered development pathway. A growing body of RA-specific PtDAs has emerged over the past decade, spanning diverse clinical settings and methods. These studies collectively demonstrate that well-designed PtDAs can significantly reduce decisional conflict, improve knowledge, and enhance clinician–patient dialogue, whereas literacy-sensitive and multimedia approaches further strengthen comprehension and engagement. At the same time, the literature highlights persistent methodological heterogeneity, variability in reporting transparency, and inconsistent outcome selection, which continue to limit cross-study comparability and cumulative learning [30,31]. Within this landscape, our contribution lies not in introducing entirely novel components but in systematically integrating evidence-supported elements within a single standards-aligned digital platform. Explicit alignment with the IPDAS scoring, UCD-11 criteria, and DEVELOPTOOLS guidance, combined with mixed-methods evaluation and iterative refinement, strengthens the transparency, reproducibility, and comparison of outcomes.

### 4.4. Limitations

Several limitations should be taken into account when interpreting the findings of this study. First, the study was conducted in a single clinical setting and involved a small convenience-based sample. Although this approach was appropriate for an early-stage, user-centered development study, it may have favored participation by individuals who were more engaged, more comfortable with digital tools, or more receptive to SDM than the broader population of patients and clinicians. As the study was conducted within a single hospital setting, the findings reflect the specific organizational routines, clinical workflows, and communication practices of that context, which may differ from those of other rheumatology services or healthcare environments. This may limit the relevance of the findings across different settings. The relatively small sample size also constrains the strength of the interpretations. While adequate for identifying key usability issues and supporting iterative refinement, it does not allow broader inferences, subgroup comparisons, or firm conclusions about the consistency of experiences across different patient or professional groups. The findings should therefore be understood as exploratory and developmental rather than representative. In addition, the study focused on the development, usability, and acceptability of the tool, rather than its longer-term decisional or clinical effects. Outcomes such as decisional conflict, value–choice concordance, consultation quality, adherence, and clinical impact were not assessed.

Finally, the PtDA was developed for the Greek healthcare context and is currently available only in Greek, although selected components have been translated to support external appraisal. While this enhances its relevance for local use, it also limits its generalizability across different linguistic, cultural, and healthcare settings without further adaptation. At the same time, the structured and theory-informed methodology underpinning its development may facilitate adaptation in other international contexts. Overall, although the IPDASi and SUS findings suggest a promising developmental foundation, the study remains exploratory. The conclusions should therefore be interpreted within the context of an early-stage study, supporting proof-of-concept and feasibility rather than broader claims of generalizability or clinical effectiveness. Future research should include larger, multicenter, and longitudinal studies to examine decisional outcomes, value–choice concordance, consultation quality, adherence, and clinical impact, as well as implementation studies exploring clinician uptake and real-world integration across diverse care settings.

## 5. Conclusions

This study represents a structured, theory-informed effort to develop a PtDA within an underrepresented healthcare context, integrating established methodological frameworks with a user-centered design approach. The integration of iterative user involvement and established evaluation criteria supported the development of a tool that shows promising methodological alignment and good perceived usability at this stage. The findings also highlight key design challenges, particularly the need to balance informational completeness with cognitive accessibility when communicating complex treatment information. Importantly, the results should be interpreted within the scope of a developmental study, reflecting usability, acceptability, and design quality rather than clinical effectiveness or generalizable impact. Future research should extend beyond early-stage evaluation to examine whether such tools can meaningfully influence decisional outcomes, support value–choice concordance, and be sustainably integrated into routine clinical practice across diverse healthcare settings.

## Figures and Tables

**Table 1:** Alpha-Testing Findings and Targeted Prototype Revisions.

Domain	Alpha Testing Feedback	Prototype 2 Revisions
Content Clarity	Patients: Clear structure and highly informative; however, complex terminology and dense statistical risk information increased cognitive burden. Clinicians: Clear structure and clinically accurate content.	Simplified terminology to enhance comprehension. Implemented a layered information structure, allowing users to access details progressively.
Risk & Benefit Presentation	Patients: Transparency was valued; however, extensive lists of side effects were perceived as overwhelming. Clinicians: Greater prominence was needed for the route of administration and expected treatment benefits, while excessive detail on rare side effects was considered likely to provoke unnecessary anxiety.	Standardized risk presentation using natural frequency formats. Reorganized the benefit–risk grid into clear, structured subsections. Reduced emphasis on rare side effects. Included clear information on time to expected symptom improvement. Enhanced the prominence of the route of administration.
Values Clarification	Patients: Supported reflection; however, some difficulty was reported in linking abstract values to concrete treatment trade-offs. Clinicians: The structured clarification of preferences enhanced the focus and efficiency of clinical consultations.	Redesigned the decision pathway (Context → Values → Comparison → Reflection). Added three subsections within a “Questions to Consider” section.
Decision Confidence & Uncertainty	Patients: Improved clarity and reassurance, while maintaining an appropriate level of residual uncertainty. Clinicians: Perceived potential to reduce decisional conflict while preserving patient autonomy.	Embedded a reflection checkpoint (“How confident do I feel about my decision?”), incorporating DCS- and SURE-informed prompts. Maintained a non-directive framing to support autonomous decision-making.
Audiovisual Support	Patients: Videos were perceived as reassuring and easier to process compared to text-heavy sections. Clinicians: Considered particularly beneficial for individuals with lower health literacy.	Shortened and segmented video content to improve usability. Added subtitles to enhance understanding. Clarified section headings to support navigation.
Usability & Navigation	Patients: Navigation was clear and user-friendly; however, some users reported minor difficulty when revisiting their responses. Clinicians: Preferred use prior to consultation and emphasized the importance of a printable output.	Added backward navigation. Optimized the summary for use during clinical consultations. Refined export and printable summary formats.
Workflow & Accessibility	Patients: Preferred completing the tool at home and explicitly requested a printable version. Clinicians: Highlighted that a printable format supports integration into routine care.	Developed a full printable version. Enabled PDF export and email functionality. Optimized the print layout for usability. Maintained a dual digital–print format.

**Table 2:** SUS Item Mean Scores by Group.

Item	SUS Item Wording	Patients (Mean ± SD)	Clinicians (Mean ± SD)	Overall (Mean ± SD)
Q1	I think that I would like to use this system frequently	3.90 ± 0.74	4.10 ± 0.69	3.98 ± 0.72
Q2	I found the system unnecessarily complex	1.90 ± 0.57	2.20 ± 0.63	2.02 ± 0.59
Q3	I thought the system was easy to use	3.90 ± 0.70	4.00 ± 0.65	3.94 ± 0.68
Q4	I think that I would need the support of a technical person	2.00 ± 0.63	2.10 ± 0.60	2.04 ± 0.61
Q5	I found the various functions were well integrated	4.60 ± 0.52	4.10 ± 0.74	4.39 ± 0.66
Q6	I thought there was too much inconsistency	2.00 ± 0.67	2.40 ± 0.70	2.16 ± 0.69
Q7	I imagine people would learn to use it very quickly	4.40 ± 0.52	4.00 ± 0.63	4.24 ± 0.58
Q8	I found the system very cumbersome to use	2.10 ± 0.57	2.00 ± 0.63	2.06 ± 0.60
Q9	I felt very confident using the system	4.00 ± 0.67	4.10 ± 0.60	4.04 ± 0.64
Q10	I needed to learn a lot of things before I could get going	1.90 ± 0.57	2.00 ± 0.63	1.94 ± 0.60

**Table 3:** User-Centered Development Reporting Based on the UCD-11 and DEVELOPTOOLS Frameworks.

Item No.	Criterion	Score (1/0)	Methodological Implementation
1	Users involved in understanding needs and context	1	Four focus groups explored decisional needs, contextual barriers, values, adherence concerns, and workflow realities prior to prototype development.
2	Users involved in designing/developing/refining prototype	1	Patients and clinicians contributed to content priorities and structural design prior to and during early prototype development.
3	Users involved in evaluating prototypes	1	Six patients and six clinicians participated in alpha testing of the web-based prototype.
4	Users asked for their opinions on the tool	1	Semi-structured interviews were conducted.
5	Users observed using the tool	1	Think-aloud protocols and teach-back techniques were utilized during alpha testing to capture real-time interaction and comprehension challenges.
6	>3 iterative cycles	1	Iterative “analyze–revise–retest” cycles occurred following focus groups, Prototype 1 development, and alpha testing refinements in preparation for beta testing.
7	Changes between cycles explicitly reported	1	Iteration-specific revisions were documented and linked to thematic findings.
8	Health professionals asked for their opinion	1	Clinicians reviewed clinical accuracy, neutrality, risk framing, and workflow feasibility during both development and alpha testing phases.
9	Health professionals consulted before first prototype	1	Clinicians participated in early focus group discussions to define the treatment comparison framework and content scope prior to initial design.
10	Health professionals consulted between initial and final prototypes	1	Clinician feedback during alpha testing informed critical refinements to comparison tables, safety framing, and workflow integration.
11	Expert panel involved	1	A multidisciplinary steering committee (rheumatologists, patient representatives, health communication expert) oversaw all development and revisions.

## Data Availability

The data supporting the findings of this study are available upon reasonable request from the corresponding author. Access to qualitative data is restricted to protect participant confidentiality.

## Supplementary Materials

The following supporting information can be downloaded at https://www.mdpi.com/article/10.3390/healthcare14080983/s1. Section S1: Focus Groups Probe Guide. Section S2: Semi-Structured Interviews Guide. Section S3: IPDASi v3 Quality Assessment of the Web-Based PtDA for R A.

## Abbreviations

The following abbreviations are used in this manuscript:

EHR	Electronic Health Record
SDM	Shared Decision Making
PtDA	Patient Decision Aid
RA	Rheumatoid Arthritis
IPDAS	International Patient Decision Aid Standards
IPDASi	International Patient Decision Aid Standards instrument
UCD	User-Centered Design
SUS	System Usability Scale
DCS	Decisional Conflict Scale
PrepDM	Preparation for Decision Making scale
OAT	Ottawa Acceptability Tool
ODSF	Ottawa Decision Support Framework
csDMARDs	Conventional Synthetic Disease-Modifying Anti-Rheumatic Drugs
bDMARDs	Biologic Disease-Modifying Anti-Rheumatic Drugs
tsDMARDs	Targeted Synthetic Disease-Modifying Anti-Rheumatic Drugs
JAK	Janus Kinase
GDPR	General Data Protection Regulation
