# Supplementary material for: Development of a Web-Based Multimedia Patient Decision Aid for Rheumatoid Arthritis: A User-Centered Design
Source: Healthcare (Basel). 2026 Apr 9;14(8):983. doi: 10.3390/healthcare14080983 (PMC13115699; doi:10.3390/healthcare14080983)
Supplement: Supplementary file 1 [file healthcare-14-00983-s001.zip › Section S1.docx]

**Focus Groups Probe Guide**

**A. Focus Group Probe Guide – Patients**

| Theme | Questions | Probes |
| --- | --- | --- |
| Theme 1: Experiences with Treatment Decision Making | 1. Can you describe your experience when you had to make decisions about your RA treatment?  2. What did you find most difficult about making these decisions? | Uncertainty, emotions, pressure, lack of clarity, time constraints |
| Theme 2: Information Needs and Gaps | 3. What kind of information did you feel you needed most when deciding about treatment? | Benefits, risks, side effects, long-term outcomes, practical implications |
| Theme 3: Values, Preferences, and Everyday Life | 5. What aspects of your daily life matter most when choosing a treatment (e.g., work, fatigue, pain, family responsibilities)?  6. How do these priorities influence your treatment choices? | Independence, quality of life, convenience, fear of side effects |
| Theme 4: Barriers and Facilitators to Decision Making | 7. What makes it easier for you to make treatment decisions?  8. What makes it harder? | Communication with doctors, trust, emotional burden, information overload |
| Theme 5: Decision Support Needs | 9. What kind of support would help you feel more confident when making treatment decisions?  10. How would an ideal decision-support tool help you? | Preparation before visits, clarity, reassurance, ability to reflect |
| Theme 6: Presentation and Information Structure | 11. How do you prefer information to be presented (short summaries vs. detailed explanations)? | Layered information, visual aids, plain language, digital vs. print |
| Theme 7: Closing Reflection | 13. If you could design a tool to help people like you decide about RA treatments, what would be most important to include? | — |

**B. Focus Group Probe Guide – Clinicians**

| Theme | Questions | | Probes |
| --- | --- | --- | --- |
| Theme 1: Clinical Decision-Making Context | | 1. How do treatment decisions for RA typically unfold in your clinical practice?  2. Which decisions do you consider most challenging for patients? | Treatment initiation, switching therapy, managing uncertainty |
| Theme 2: Perceived Patient Decisional Needs | | 3. Where do patients most commonly struggle when making treatment decisions?  4. What misunderstandings or concerns do you frequently encounter? | Risks vs. benefits, expectations, fear of adverse effects |
| Theme 3: Values and Preference Sensitivity | | 5. How do patients’ personal values and life circumstances influence treatment decisions?  6. How easy or difficult is it to elicit these values during routine consultations? | Time constraints, communication challenges, variability across patients |
| Theme 4: Barriers and Facilitators to Shared Decision Making | | 7. What facilitates shared decision making in RA care?  8. What are the main barriers? | Time pressure, information complexity, health literacy, system constraints |
| Theme 5: Information Priorities for a PtDA | | 9. What information should absolutely be included in a PtDA for RA?  10. What information might be unnecessary or overwhelming for patients? | Clinical outcomes, side effects, uncertainty, comparative framing |
| Theme 6: Integration into Clinical Workflow | | 11. At what point in the care pathway would a PtDA be most useful?  12. How could such a tool realistically fit into routine practice? | Pre-visit use, in-consultation support, printed vs. digital formats |
| Theme 7: Layered Information and Design Considerations | | 13. How do you view the idea of layered information (basic content upfront, details on demand)?  14. Would this approach support patients with different levels of health literacy? | Cognitive load, personalization, usability |
| Theme 8: Closing Reflection | | 15. From your perspective, what would make a PtDA genuinely useful and acceptable in RA care? | — |
