# Supplementary material for: Development of a Web-Based Multimedia Patient Decision Aid for Rheumatoid Arthritis: A User-Centered Design
Source: Healthcare (Basel). 2026 Apr 9;14(8):983. doi: 10.3390/healthcare14080983 (PMC13115699; doi:10.3390/healthcare14080983)
Supplement: Supplementary file 1 [file healthcare-14-00983-s001.zip › Section S2.docx]

**Semi-Structured Interviews Guide**

1. **Semi-Structured Interview Guide- Patients**

| **Domain** | **Questions** |
| --- | --- |
| Domain 1: Content (Clarity, comprehensibility, and support for understanding options) | 1. What were your first impressions of the PDA?  2. How easy or difficult was it to understand the information?  3. Do you feel you now understand:  • What should I consider when making this decision  • What matters most to me section  • Factors that influence my decision  • The treatment options?  • The benefits of each option?  • The risks and side effects of each option?  • The contradictions of each option  • The combination of the options  4. Was the information presented in a balanced and neutral way?  5. Were any parts confusing, overwhelming, or unclear?  6. Was the overall length of the PDA: Too long -Too short -About right  7. The amount of information was: Too much -Too little - About right  8. Do you feel any important information was missing? |
| Domain 2: Effectiveness in Supporting Decision Making (Values clarification, reduction of uncertainty, confidence, preparation for consultation) | 9. Did the section on what is important to you help you clarify your personal priorities?  10. Did it help you weigh the benefits and risks according to what matters most to you?  11. Did comparing treatments side-by-side help you think about the pros and cons?  12. After completing the PDA, do you feel clearer about the best choice for you?  13. Do you feel more certain or less uncertain than before?  14. Does the decision you are considering reflect what is important to you?  15. Do you feel more confident that you can:   - Ask questions? - Express your concerns? - Participate actively in the decision?   16. Did the PDA help you prepare for discussing treatment options with your doctor?  17. Did it help you identify questions you would like to ask? |
| Domain 3: Usability (Navigation, structure, accessibility, digital experience) | 18. Was the structure of the PDA logical and easy to follow?  19. Was it easy to navigate between sections?  20. Did the comparison cards visuals, help you understand the information?  21. Did videos, help you understand the information?  22. Was any page visually dense or difficult to process?  23. Did you experience any technical difficulties??  24. Would you feel comfortable using this tool again? |
| Closing Questions | 25. Overall, how useful was the PDA for you?  26. Would you recommend it to other patients?  27. What would you improve?  “Is there anything else you would like to share?” |

1. **Semi-Structured Interview Guide- Clinicians**

| **Domain** | **Questions** |
| --- | --- |
| Domain 1: Content (Clinical accuracy, appropriateness, balance, and relevance) | 1. What were your initial impressions of the PDA?  2. Is the clinical information accurate and up to date?  3. Is the level of detail appropriate for patients with moderate-to-severe RA?  4. Is the presentation of benefits and risks balanced and neutral?  5. Does the content reflect real-world therapeutic considerations?  6. Is the comparison of treatment options clinically appropriate? |
| Domain 2: Effectiveness in Supporting Decision Making (Support for shared decision making and decision quality) | 7. To what extent does the PDA support shared decision making in RA?  8. Does the “Questions to consider ” section help patients articulate meaningful values during consultation?  9. Does the treatment comparison section facilitate patient understanding of trade-offs?  10. Do you think the PDA could reduce decisional conflict?  11. Could it improve patients’ confidence and clarity about their choice?  12. Does it help align treatment decisions with patient priorities?  13. Do you think it may enhance the quality of patient–clinician dialogue?  14. Could it contribute to better adherence or more realistic expectations? |
| Domain 3: Usability and Integration into Clinical Practice (Workflow fit, feasibility, accessibility, endorsement) | 15. How feasible would it be to integrate this PDA into routine rheumatology practice?  16. At what point in the care pathway would it be most useful (before, during, or after consultation)?  17. Would it fit within typical consultation time constraints?  18. Do you foresee implementation barriers (time, workflow, patient literacy, digital skills)?  19. How would you rate the usability and structure of the PDA?  20. Is the design appropriate for patients with varying levels of health and digital literacy?  21. Are the audiovisual components clinically useful?  22. Would you feel comfortable recommending this PDA to your patients? |
| Closing | 23. Overall, how acceptable is this PDA for use in RA care?  24. Would you recommend its broader use?  25. What key improvements would you suggest before wider implementation?  “Is there anything else you would like to add?” |
