# Supplementary material for: Development of a Web-Based Multimedia Patient Decision Aid for Rheumatoid Arthritis: A User-Centered Design
Source: Healthcare (Basel). 2026 Apr 9;14(8):983. doi: 10.3390/healthcare14080983 (PMC13115699; doi:10.3390/healthcare14080983)
Supplement: Supplementary file 1 [file healthcare-14-00983-s001.zip › Section S3.docx]

**IPDASi v3 Quality Assessment of the Web-Based PtDA for RA**

The web-based PtDA was assessed using the IPDASi v3 framework. Overall, the tool meets most of the key quality criteria, particularly in presenting clear, balanced information about treatment options and supporting patients in reflecting on what matters most to them. Areas requiring further strengthening relate mainly to formal reporting elements—such as documenting uncertainty, update policy, and published evaluation outcomes—rather than to the core structure or content of the decision-support process.

| N | Category | IPDASi Item | Score | Criteria Met |
| --- | --- | --- | --- | --- |
| 1 | INFO | The decision support technology describes the health condition or problem (intervention, procedure or investigation) for which the index decision is required | 4 | Rheumatoid arthritis is clearly described (nature, symptoms, and consequences). |
| 2 | INFO | The decision support technology describes the decision that needs to be considered (the index decision) | 4 | The treatment decision is explicitly defined. |
| 3 | INFO | The decision support technology describes the options available for the index decision | 4 | All pharmacological treatment options are clearly presented. |
| 4 | INFO | The decision support technology describes the natural course of the health condition or problem, if no action is taken. | 3 | Disease progression is described, but not quantitatively detailed. |
| 5 | INFO | The decision support technology describes the positive features (benefits or advantages) of each option | 4 | Benefits are comprehensively described for each therapy. |
| 6 | INFO | The decision aid describes negative features (harms, side effects or disadvantages) of each option. | 4 | Side effects are described for each therapy. |
| 7 | INFO | The decision support technology makes it possible to compare the positive and negative features of the available options. | 4 | A structured side-by-side comparison is integrated within the tool. |
| 8 | INFO | The decision support technology shows the negative and positive features of options with equal detail (for example using similar fonts, order, and display of statistical information). | 4 | Benefits, symptom improvement, and side effects are consistently presented across therapies. |
| 9 | PROB | The decision support technology provides information about outcome probabilities associated with the options (i.e. the likely consequences of decisions) | 4 | Numerical probabilities are presented within the benefits and side effects sections. |
| 10 | PROB | The decision support technology specifies the defined group (reference class) of patients for which the outcome probabilities apply. | 2 | The target population is not explicitly defined alongside the probabilities. |
| 11 | PROB | The decision support technology specifies the event rates for the outcome probabilities (in natural frequencies). | 4 | Event rates are presented in natural frequencies (e.g., “1 in 10”) for both benefits and harms. |
| 12 | PROB | The decision support technology specifies the time period over which the outcome probabilities apply. | 4 | Time horizons are clearly stated for therapeutic outcomes (e.g., 4–12 weeks). |
| 13 | PROB | The decision support technology allows the user to compare outcome probabilities across options using the same denominator and time period. | 4 | Comparable metrics are applied across option. |
| 14 | PROB | The decision support technology provides information about the levels of uncertainty around event or outcome probabilities (e.g. by giving a range or by using phrases such as “our best estimate is…”) | 1 | No explicit reporting of statistical uncertainty or variability is provided. |
| 15 | PROB | The decision support technology provides more than one way of viewing the probabilities (e.g. words, numbers, and diagrams). | 4 | Probabilities are presented using text, natural frequencies, and icon arrays. |
| 16 | PROB | The decision support technology provides balanced information about event or outcome probabilities to limit framing biases. | 3 | A consistent approach is used, although complementary framing is not systematically applied. |
| 17 | VALUES | The decision support technology describes the features of options to help patients imagine what it is like to experience the physical effects. | 4 | The physical impact of treatments is clearly described. |
| 18 | VALUES | The decision support technology describes the features of options to help patients imagine what it is like to experience the psychological effects. | 4 | Emotional implications are addressed. |
| 19 | VALUES | The decision support technology describes the features of options to help patients imagine what it is like to experience the social effects. | 4 | Social and daily-life impacts are discussed. |
| 20 | VALUES | The decision support technology asks patients to think about which positive and negative features of the options matter most to them. | 4 | A structured values clarification component is included. |
| 21 | GUIDE | The decision support technology provides a step-by-step way to make a decision. | 4 | A clear three-step decision pathway is implemented. |
| 22 | GUIDE | The decision support technology includes tools like worksheets or lists of questions to use when discussing options with a practitioner. | 4 | A consultation checklist is provided for clinical encounters. |
| 23 | DEV | The development process included finding out what clients or patients need to prepare them to discuss a specific decision | 4 | Patient needs were assessed during development. |
| 24 | DEV | The development process included finding out what health professionals need to prepare them to discuss a specific decision with patients | 4 | Clinician input was incorporated during development. |
| 25 | DEV | The development process included expert review by clients/patients not involved in producing the decision support technology | 4 | External patient stakeholders reviewed draft prototypes. |
| 26 | DEV | The development process included expert review by health professionals not involved in producing the decision aid. | 4 | External health professionals reviewed draft prototypes and materials. |
| 27 | DEV | The decision support technology was field tested with patients who were facing the decision. | 4 | Alpha and beta testing were conducted with patients. |
| 28 | DEV | The decision support technology was field tested with practitioners who counsel patients who face the decision. | 4 | Alpha and beta testing were conducted with clinicians. |
| 29 | EVID | The decision support technology (or associated documentation) provides citations to the studies selected. | 2 | A general reference to the literature is provided; however, explicit study-level citations are not listed. |
| 30 | EVID | The decision support technology (or associated documentation) describes how research evidence was selected or synthesized. | 3 | The literature review process is described; however, the absence of a detailed PRISMA flow chart limits transparency. |
| 31 | EVID | The decision support technology (or associated documentation) provides a production or publication date. | 4 | The publication year is clearly stated. |
| 32 | EVID | The decision support technology (or associated documentation) provides information about the proposed update policy. | 1 | No explicit update or revision policy is reported. |
| 33 | EVID | The decision support technology (or associated documentation) describes the quality of the research evidence used. | 2 | Evidence sources are referenced; however, no formal grading or quality assessment framework is specified. |
| 34 | DISCL | The decision support technology (or associated technical documentation) provides information about the funding used for development. | 4 | The funding source is explicitly disclosed (unrestricted medical grant). |
| 35 | DISCL | The decision support technology includes author/developer credentials or qualifications. | 4 | Academic and institutional credentials are clearly stated. |
| 36 | LANG | The decision support technology (or associated documentation) reports readability levels (using one or more of the available scales). | 1 | No formal readability metric is reported. |
